# Spatial Variation in Bacterioplankton Communities in the Pearl River, South China: Impacts of Land Use and Physicochemical Factors

**DOI:** 10.3390/microorganisms8060814

**Published:** 2020-05-29

**Authors:** Lei Zhou, Weiyuan Chen, Jijia Sun, Li Liu, Xiande Huang

**Affiliations:** 1 Joint Laboratory of Guangdong Province and Hong Kong Region on Marine Bioresource Conservation and Exploitation, College of Marine Sciences, South China Agricultural University, Guangzhou 510642, China; zhoulei@scau.edu.cn (L.Z.); cheninmarine@stu.scau.edu.cn (W.C.); jjsun@scau.edu.cn (J.S.); 2 Guangdong Laboratory for Lingnan Modern Agriculture, Guangzhou 510642, China

**Keywords:** bacterioplankton, spatial variation, land use, physicochemical factors, Pearl River

## Abstract

River ecosystems are critical for human and environmental health, with bacterioplankton playing a vital role in biogeochemical cycles. Unveiling the spatial patterns of bacterioplankton communities in relation to environmental factors is important for understanding the processes of microbial variation and functional maintenance. However, our understanding of the correlations among bacterioplankton communities, physicochemical factors, and land use, especially in large rivers affected by intensive anthropogenic activities, remains relatively poor. Here, we investigated the bacterioplankton communities in July 2018 in three main tributaries of the Pearl River, i.e., Beijiang, Xijiang, and Pearl River Delta, based on 16S rRNA high-throughput sequencing. Results showed that the most dominant phyla, Proteobacteria, Actinobacteria, Cyanobacteria, and Planctomycetes accounted for 33.75%, 22.15%, 11.65%, and 10.48% of the total abundance, respectively. The bacterioplankton communities showed remarkable differences among the three tributaries in terms of composition, structure, diversity, and predictive functional profiles. Mantel and partial Mantel tests revealed that the bacterioplankton communities were affected by physicochemical variables (*p* < 0.01) and land use (*p* < 0.01). Redundancy analysis identified specific conductivity, dissolved oxygen, agricultural land, ammonium, urban land, and water transparency as the dominant environmental factors influencing the bacterioplankton communities in the Pearl River. Variation partitioning analysis indicated that both physicochemical factors and land use had direct effects on the bacterioplankton community, and that land use may also shape bacterioplankton communities through indirect effects of physicochemical factors on riverine ecosystems. This study provides fundamental information on the diversity, spatial patterns, and influencing factors of bacterioplankton communities in the Pearl River, which should enhance our understanding of how such communities change in response to environmental gradients and anthropogenic activities.

## 1. Introduction

The microbial community is a key component of aquatic ecosystems and plays an important role in biogeochemical cycling processes such as ammonia oxidation, nitrification, sulfate reduction, and methane production [1,2,3,4]. Unveiling the spatial patterns of microbial communities in relation to environmental factors is important for understanding the processes of microbial variation and functional maintenance [5,6]. Planktonic microorganisms are highly dynamic within aquatic systems [7,8,9] and variations in bacterioplankton communities in terms of composition, diversity, and function may occur in response to environmental change [10,11].

River ecosystems are critical for human and ecological health. They are a major source of drinking water and provide irrigation for agriculture, habitat for fish, and space for recreation. Rivers also offer important freshwater habitats for microorganisms. Bacterioplankton communities exhibit complex responses to environmental stresses and disturbances, as manifested by changes at both the population and community levels [12,13]. Many studies have attempted to elucidate the distribution patterns and driving factors shaping bacterioplankton communities in river ecosystems. Such studies have shown that the bacterioplankton community structure can be strongly influenced by local environmental conditions within the water channel, such as temperature [13] and nutrient [14], suspended solids [4], and dissolved oxygen levels [15]. In addition, as lotic river water is under constant turnover, free-floating microbial communities at fixed geographic locations are composed of immigrant microorganisms from the planktonic community upstream and surrounding non-planktonic communities. Surrounding inputs can affect microbial plankton directly, e.g., through the influx of organisms, and indirectly, e.g., via changes in conditions leading to shifts in competitive advantages and thus microbial communities. As such, catchment geography and land use, e.g., agriculture and urbanization, are important drivers of variations in bacterioplankton communities of lotic ecosystems [16,17]. However, despite increasing knowledge of bacterioplankton in river ecosystems, our understanding of the associations among bacterioplankton communities, physicochemical factors, and land use, especially in large rivers impacted by intensive anthropogenic activities, remains relatively limited.

The Pearl River is the largest river in southern China, stretching some 2400 km in length [18]. The river is used for a wide range of purposes (e.g., agriculture, industry, transport, and recreation) and is an important drinking water source for many surrounding cities. Thus, the Pearl River area experienced considerable anthropogenic disturbance due to remarkable industrial and agricultural development along with rapid population growth [19]. For example, the Pearl River Delta, is one of China’s most industrialized regions, with a population of more than 50 million and annual wastewater production of about 9 × 10^9^ m^3^, including 7 × 10^9^ and 2 × 10^9^ m^3^ of domestic and industrial wastewater, respectively [20]. Such waste can have a negative impact on the biotic and abiotic components of riverine ecosystems [21]. Although bacterioplankton community dynamics in the Pearl River Estuary are well studied [22,23,24,25], our understanding of bacterioplankton communities in the Pearl River and their responses to environmental gradients and land use activity is still lacking.

In this study, we analyzed the bacterioplankton communities in three main tributaries of the Pearl River in July 2018, a typical flood season, using 16S rRNA high-throughput sequencing. We aimed to (1) characterize the composition, diversity, and spatial patterns of bacterioplankton communities in the Pearl River and (2) elucidate the roles of physicochemical factors and land use in shaping these communities. To the best of our knowledge, this is the first study to report on the effects of land use and physicochemical factors on bacterioplankton communities in the Pearl River, which should enhance our understanding of how aquatic ecosystems change and function in response to environmental gradients and anthropogenic activities.

## 2. Materials and Methods

### 2.1. Study Site and Sampling

**Figure 1:**
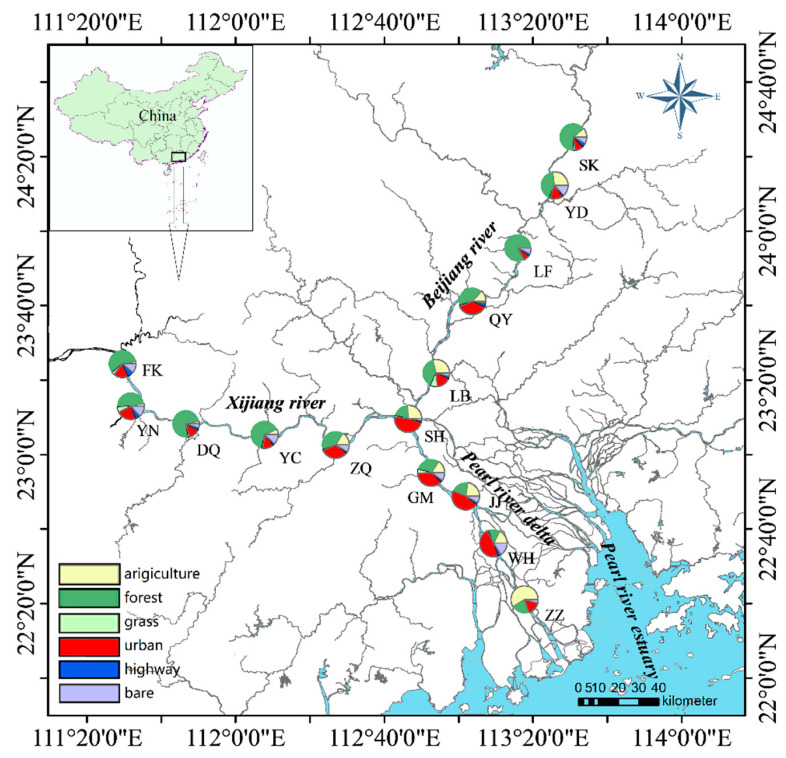
Location of the Pearl River study sites. Pie plot shows land use cover (%).

A total of 30 water samples were collected in July 2018 from 15 sites (FK, YN, DQ, YC, ZQ for Xijiang River (XJ); SK, YD, LF, QY, LB for Beijing River (BJ); and SH, GM, JJ, WH, ZZ for Pearl River Delta (PRD) (Figure 1) along the Pearl River in southern China. At each site, duplicate surface water samples (approximately 30–50 m apart) were obtained. All the water samples were collected at 0.5 m depth to minimize the error caused by sampled depth. The water samples (500 mL) were filtered for 16S rRNA sequencing through 0.2 μm pore polycarbonate membranes (Millipore, MA, USA). Another 1000 mL of water was used for physicochemical analyses.

### 2.2. Physicochemical Analyses and Land Use Data

Physicochemical variables, including dissolved oxygen (DO), temperature, salinity, pH, total dissolved solids (TDS), specific conductivity (SpC), and oxidation-reduction potential (ORP), were measured in situ with a YSI multiparameter probe (YSI Proplus, Yellow Springs, OH, USA). Water transparency (cm) was measured by a Secchi disk (SunVote, Changsha, China) (20 cm diameter). Ammonium (NH_4_-N), nitrate (NO_3_-N), nitrite (NO_2_-N), total phosphorus (TP), phosphate (PO_4_-P), chlorophyll*a*, and suspended solids (SS), were measured in the laboratory following standard protocols [26,27].

Satellite datasets from the Landstat 8 OLI database (Available online: https://landsat.gsfc.nasa.gov) (2016–2018) were used to determine land use cover. The satellite images were corrected and stitched in ENVI 5.3 (ITT Visual Information Solutions, Boulder, CO, USA), and then they classified into land use types using knowledge-based supervised and maximum likelihood classification methods. Land use was classified into agricultural land, forestland, grassland, urban land, highway, and bare soil. The 1:100,000 scale map of land use was then interpreted. ArcGIS 10.2.2 (ESRI, Redlands, CA, USA) was applied to extract land-use percentages using a 10 km × 2 km buffer zone upstream of the sampling points.

### 2.3. DNA Extraction, PCR Amplification, and 16S rRNA Sequencing

DNA was extracted from all samples using the HiPure Stool DNA Kits (Magen, Guangzhou, China). The 16S rDNA V3-V4 hypervariable region was amplified using primers 341F (CCTACGGGNGGCWGCAG) and 806R (GGACTACHVGGGTATCTAAT). After being quantified with QuantiFluorTM fluorometer, purified amplicons by PCR were pooled in equimolar amounts and paired ends were sequenced (2 × 250) on an Illumina HiSeq2500 platform according to the standard protocols at Gene Denovo Biological Technology Co. Ltd. (Guangzhou, China). The 16S high-throughput sequencing raw data was uploaded in the NCBI Sequence Read Archive with accession no. PRJNA579235.

### 2.4. Processing of Sequencing Data

The raw reads were filtered using FASTP. Paired-end clean reads were then merged using FLASH v. 1.2.11 [28]. Noisy sequences of the raw tags were further filtered by the QIIME v. 1.9.1 [29] to obtain clean tags [30]. The clean tags were then subjected to reference-based chimera checking and removal using the UCHIME algorithm to obtain effective tags. The effective tags were clustered into operational taxonomic units (OTU) of ≥97% similarity using the UPARSE [31] pipeline. The representative sequence within each cluster was selected from the tag sequence with the highest abundance and then classified into taxonomic groups by a naive Bayesian model using the Ribosomal Database Project (RDP) classifier [32] based on the SILVA [33] database.

### 2.5. Statistical Analysis

Physicochemical factors and land use were analyzed by principal component analysis (PCA) to identify potential spatial patterns in the different tributaries.

Alpha diversity, including Good’s coverage, Abundance-based Coverage Estimator (ACE), Chao1, Simpson, and Shannon indices, were calculated via QIIME. Differences in diversity indices among the different tributaries were examined with Kruskal–Wallis tests for global comparison and Wilcoxon tests for pairwise comparisons (*p* < 0.05).

Nonmetric multidimensional scaling (NMDS) and analysis of similarity (ANOSIM) were performed to compare bacterioplankton communities based on the Bray–Curtis dissimilarity index using the Vegan package in R [34]. Linear discriminant analysis (LDA) and linear effect size (LEfSe) analyses were used to detect taxa with differential abundance among the three tributaries with the Galaxy online tool (http://huttenhower.sph.harvard.edu/lefse/).

The Mantel test was used to examine the correlation between the environment (e.g., local physicochemical factors and land use) and bacterioplankton communities, and a partial Mantel test was used to determine the relationship between a certain type of factor (local physicochemical factors or land use) and bacterioplankton community, controlling for the effects of other factors (land use or local physicochemical factors). The Mantel and partial Mantel tests were conducted with the Vegan package [34]. Redundancy analysis (RDA) was used to determine associations between bacterioplankton communities and environmental factors. Variation partitioning analysis (VPA) was performed to determine important variables (local physicochemical factors versus land use), how much variation each explained, and the size of their shared effects. The Monte Carlo permutation test (permutations = 499) was used to assess the statistical significance of these relationships. Both RDA and VPA analyses were performed using CANOCO 5.0 software [35]. 

Functional changes in the bacterioplankton communities among the different tributaries were predicted using Phylogenetic Investigation of Communities by Reconstruction of Unobserved States (PICRUSt) [36]. Rarefied 16S rRNA copy numbers were used to reconstruct the metagenome functional genes, which were further classified via KEGG categories at levels 1, 2, and 3 [37]. Multifunctional diversity using Shannon H as a diversity index was calculated based on the KEGG category abundances following Peter and Sommaruga [38]. Aggregated boosted tree analysis was performed to assess the relative influence of physicochemical factors and land use on multifunctional diversity, using the gbmplus package [39].

## 3. Results

### 3.1. Environmental Variables

**Figure 2:**
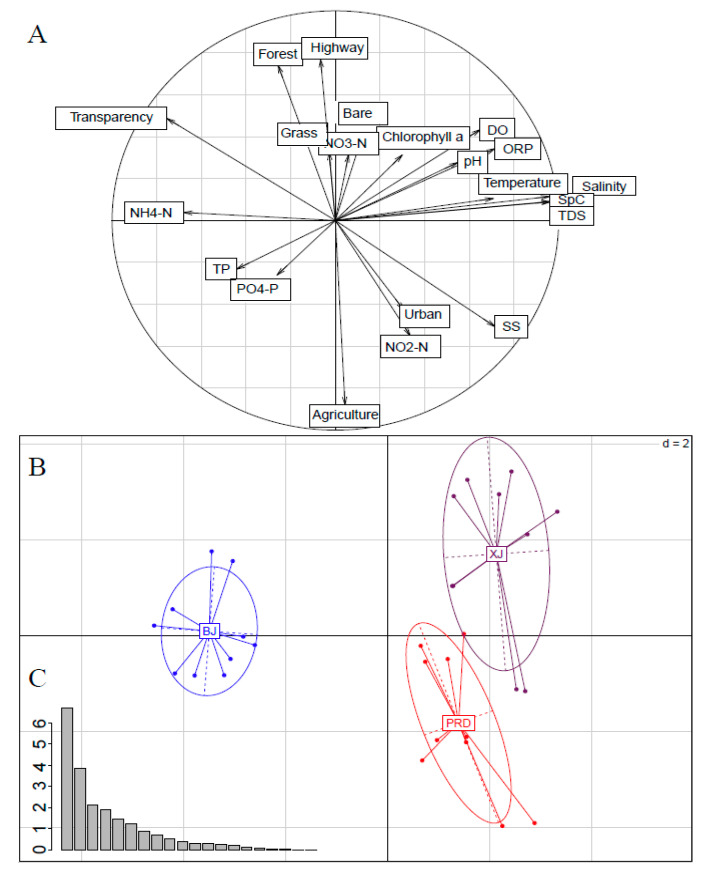
Principal component analysis (PCA) results for environmental variables in the Pearl River. (**A**) Vector plot indicating correlations among environmental variables and their scores on axis 1 and 2. (**B**) Multivariate analyses of environmental variables using a scatter diagram, with each tributary presented as an ellipsoid. (**C**) Bar plot showing eigenvalues in PCA. BJ, Beijiang; XJ, Xijiang; and PRD, Pearl River Delta. The abbreviations of environmental variables are defined in Materials and Methods.

Spatial variations in 15 physicochemical factors and six land use types were analyzed by PCA to identify differences among tributaries (Figure 2). The first two principal components described 31.82% and 18.44% of total environmental variation, respectively (Figure 2B). Axis 1 showed a gradient of physicochemical parameters and axis 2 showed a gradient of land use (Figure 2C). Axis 1 was highly positively correlated with salinity (SpC, TDS), SS, and ORP, and negatively associated with water transparency and NH_4_-N. Axis 2 was highly positively correlated with highways and forests, and negatively associated with agricultural land.

Both XJ and PRD were close to each other, but distant from BJ (Figure 2A). BJ was prominently separated from XJ and PRD by the first PCA axis, whereas XJ and PRD were further differentiated by the second axis. Water transparency and NH_4_N were relatively higher in BJ than in XJ and PRD, whereas DO and ORP were relatively higher in XJ than in BJ and PRD. PRD had relatively higher values for agricultural land, urban land, SS, and NO_2_N, but lower values related to water transparency, forests and highways.

### 3.2. Bacterioplankton Community Composition, Diversity, and Spatial Variations

**Figure 3:**
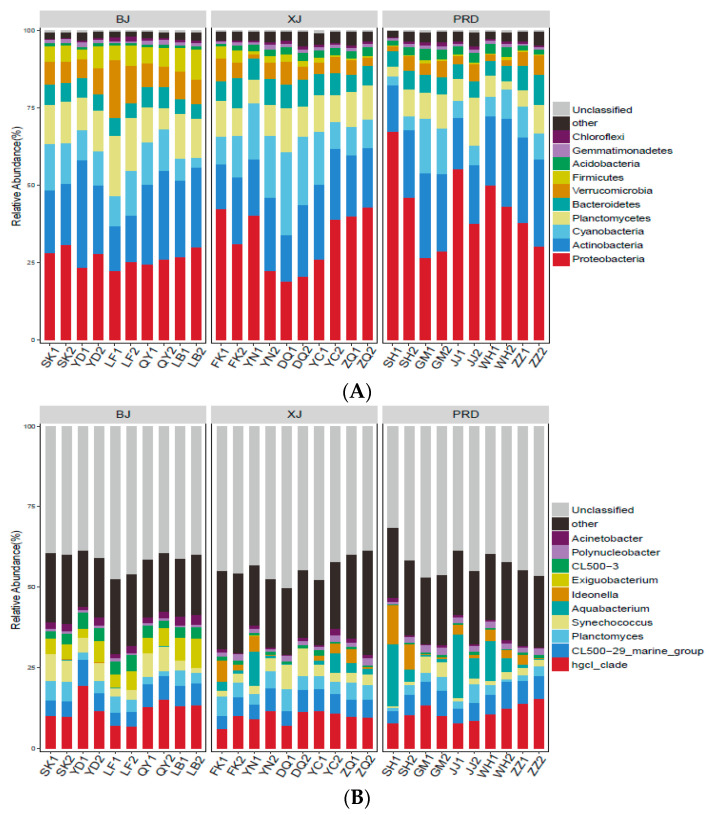
Composition of bacterioplankton community at the phylum (**A**) and genus levels (**B**) across all samples. Only the top 10 taxa with the largest mean relative abundance are shown. BJ, Beijiang; XJ, Xijiang; and PRD, Pearl River Delta.

A total of 1,686,856 high-quality sequences were generated from all samples. Rarefaction curves for all samples demonstrated a plateau (Figure S1), indicating that the number of sequences analyzed sufficiently represented bacterial diversity. Further, Good’s coverage values for all samples ranged from 0.979 to 0.989, confirming the completeness of sequencing. The number of sequences per sample ranged from 41,800 to 76,447, with an average of 56,229 sequences per sample. For comparison, operational taxonomic unit (OTU) abundances were normalized to 41,800 sequences in subsequent analyses. Of the classifiable sequences, 53 phyla were identified. The most dominant groups, Proteobacteria, Actinobacteria, Cyanobacteria, Planctomycetes, Bacteroidetes, Verrucomicrobia, Firmicutes, Acidobacteria, Gemmatimonadetes, and Chloroflexi, accounted for 33.75%, 22.15%, 11.65%, 10.48%, 6.34%, 5.85%, 2.78%, 1.91%, 1.01%, and 0.77% of total abundance, respectively (Figure 3A). At the genus level, 747 genera were identified. The most abundant groups were *hgcI_clade*, *CL500-29_marine_group*, *Planctomyces*, *Synechococcus*, *Aquabacterium*, *Ideonella*, *Exiguobacterium*, *CL500-3*, *Polynucleobacter,* and *Acinetobacter*, which accounted for 10.81%, 6.03%, 3.84%, 3.71%, 3.25%, 1.92%, 1.86%, 1.74%, 1.33%, and 1.23% of total bacterioplankton community, respectively (Figure 3B)

**Figure 4:**
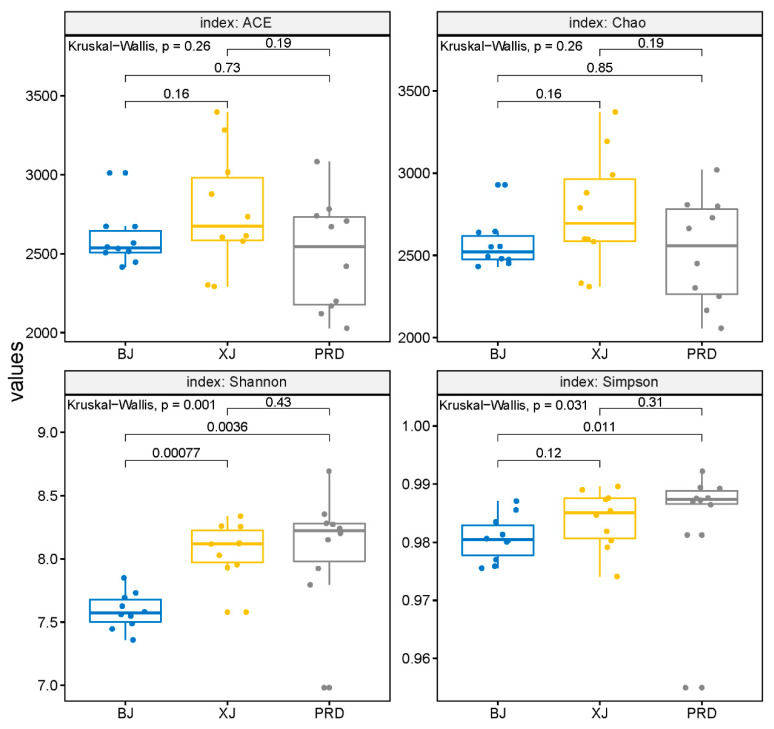
Comparison of alpha diversities, including Abundance-based Coverage Estimator (ACE), Chao, and Shannon and Simpson indices among different Pearl River tributaries. BJ, Beijiang; XJ, Xijiang; and PRD, Pearl River Delta.

For the diversity indices, the number of OTUs ranged from 1597 to 2450, with an average of 1954. The Shannon and Simpson indices ranged from 6.98 to 8.69 and 0.95 to 0.99, respectively. The Chao and ACE indices ranged from 2056.7 to 3372.4 and 2028.1 to 3397.1, respectively. Kruskal-–Wallis tests demonstrated no significant differences in species richness indices (Chao and ACE) among the three tributaries, whereas diversity indices (Shannon and Simpson) showed significant spatial variation (Figure 4). The Shannon and Simpson index values were higher in PRD than those in BJ, whereas no significant differences existed between PRD and XJ. In addition, XJ showed higher diversity than BJ, but only for the Shannon index.

**Figure 5:**
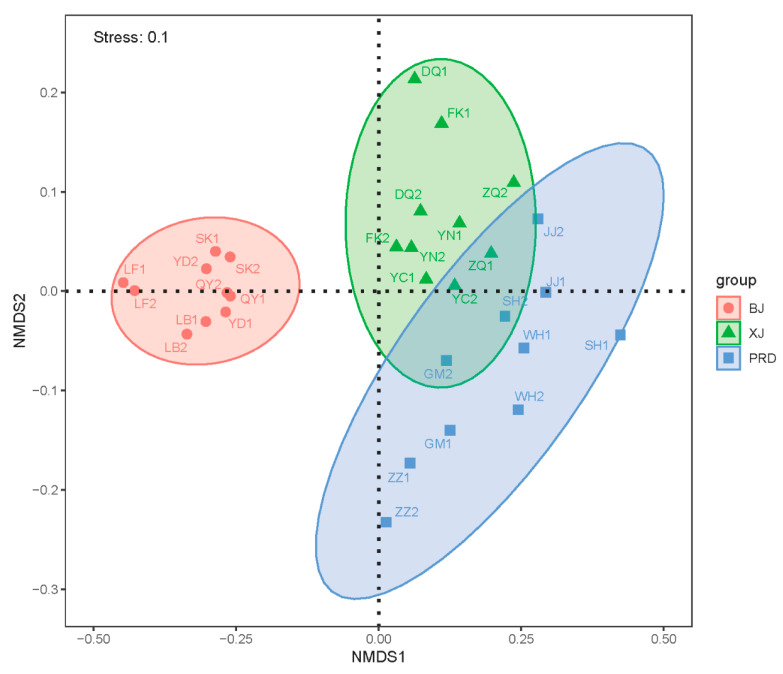
Nonmetric multidimensional scaling (NMDS) plots of bacterioplankton communities along the Pearl River based on the Bray–Curtis distance. Ellipses represent 95% confidence interval.

Based on the NMDS and ANOSIM analyses, the samples within the three tributaries showed significant clustering (Figure 5; Table 1; ANOSIM, *p* < 0.01). The BJ group was separated from the other groups, whereas XJ and PRD demonstrated partial overlap. These spatial patterns were consistent with environmental factors (Figure 2).

**Figure 6:**
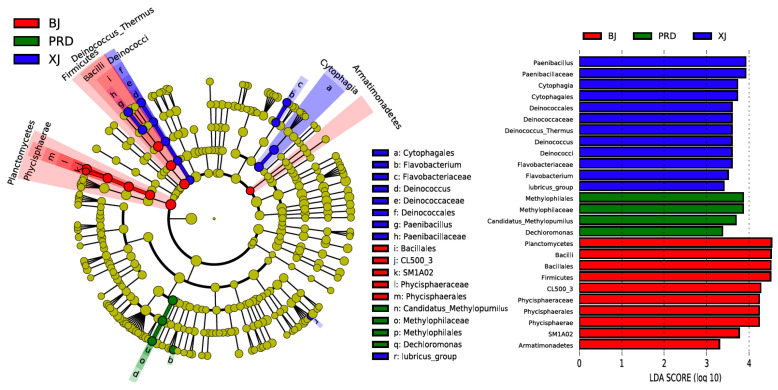
Linear discrimination analysis (LDA) and linear effect size (LEfSe) analysis identified most differentially abundant taxa (LDA values > 3) across the Pearl River tributaries. Differentially abundant taxa of each tributary are distinguished by different colors. Taxa without significant difference are uniformly yellow. Radiating circles from inside to out represent taxonomic levels from phylum to genus.

LEfSe analysis was used to determine indicator taxa associated with the three tributaries. Across phylum to genus, 12, 4, and 10 indicators were identified for XJ, PRD, and BJ groups, respectively. Some lineages belonging to Proteobacteria, such as Methylophilales (order), Methylophilaceae (family), Candidatus*_Methylopumilus* (genus), and *Dechloromonas* (genus), were identified as potential biomarkers for PRD (Figure 6). Genera *Deinococcus*, *Paenibacillus*, *Flavobacterium,* and *lubricus*_group were significantly enriched in XJ, whereas *SM1A02* and *CL500_3* showed high relative abundance in BJ.

### 3.3. Effects of Physicochemical Variables and Land Use on Bacterioplankton Communities

**Figure 7:**
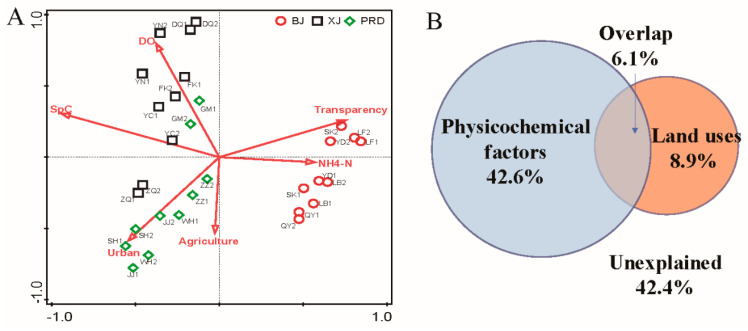
Effects of physicochemical variables and land use on bacterioplankton communities. (**A**) Redundancy analysis (RDA) ordination showing associations between bacterioplankton and environmental factors in the Pearl River. (**B**) Variation partitioning of bacterioplankton communities by physicochemical variables and land use.

The Mantel tests indicated that environmental factors significantly shaped the bacterioplankton communities (Mantel test: *r* = 0.638; *p* = 0.001). Partial Mantel tests revealed that the bacterioplankton communities were affected by both physicochemical variables (partial Mantel test: *r* = 0.618; *p* = 0.001) and land use (partial Mantel test: *r* = 0.225; *p* = 0.001). Forward selection and Monte Carlo permutation (499 iterations) of RDA showed that environmental variables SpC (*F* = 14.9; *p* = 0.002), DO (*F* = 4.4; *p* = 0.002), agricultural land (*F* = 2; *p* = 0.006), NH_4_-N (*F* = 2; *p* = 0.004), urban land (*F* = 1.9; *p* = 0.014), and water transparency (*F* = 1.8; *p* = 0.016) significantly influenced the Pearl River bacterioplankton communities (Figure 7A). The first and second axes modeled 39.52% and 10.04% of total variance for bacterioplankton communities, respectively. We conducted VPA to determine which variables (physicochemical variables versus land use) were important and how much variance each explained alone or in combination. Physicochemical variables and land use together explained 57.6% of total variation (Figure 7B; *F* = 3; *p* = 0.002). The conditional effect of physicochemical variables was 42.6% (*F* = 2.6; *p* = 0.002). The conditional effect of land use was 8.9% (*F* = 1.6; *p* = 0.02). The shared effect of these two groups was 6.1%. In total, 42.4% of variation remained unexplained. 

**Figure 8:**
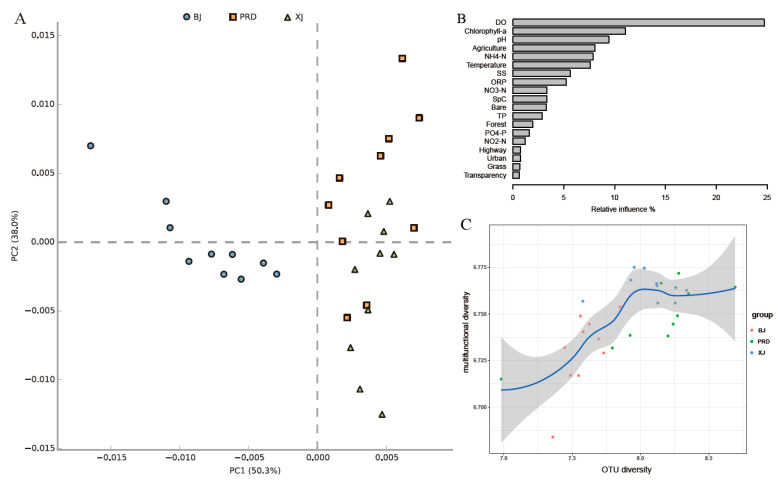
Predicted functional profiles and multifunctional diversities of bacterioplankton communities in the Pearl River. (**A**) Principal component analysis (PCA) of predicted bacterioplankton community function. (**B**) Relative influence (%) of environmental factors on multifunctional diversity via aggregated boosted tree models. (**C**) Relationship between operational taxonomic units (OTU) diversity and multifunctional diversity. Blue line indicates fit between OTU diversity (Shannon) and multifunctional diversity. Gray shadow under blue line represents 95% confidence intervals.

For predicted functional profiling, Kruskal–Wallis tests indicated that there were significant differences in all predicted KEGG level 2 pathways among the three tributaries except for “Cell Growth and Death” within Cellular Processes and “Digestive System” within Organismal Systems (Table S1). The functional variations among the three tributaries showed a similar spatial pattern as the bacterioplankton communities, that is, BJ was separated from the two other tributaries, whereas the functions of XJ and PRD showed partial overlap (Figure 8A).

Analyses based on aggregated boosted tree models were used to interpret the relative importance of physicochemical factors and land use on multifunctional diversity. Results indicated that DO was the major factor affecting multifunctional diversity, accounting for 24.73% of relative influence (Figure 8B), with Chlorophyll*a*, pH, agricultural land, and NH_4_-N accounting for 11.06%, 9.43%, 8.06%, and 7.88%, respectively. The top five factors explained over half of the relative influence. The fitted line showed that with increasing OTU diversity (Shannon index), multifunctional diversity also increased, but when OTU diversity approached 8, multifunctional diversity reached saturation, indicating functional redundancy within the bacterioplankton communities (Figure 8C).

## 4. Discussion

Microbial communities are fundamental in the functioning of river ecosystems. The present study evaluated the bacterioplankton community in a large subtropical river that is highly modified by human activities. We revealed that the three tributaries formed distinct bacterioplankton communities, which were, in turn, significantly influenced by physicochemical variables and land use.

The predominant bacterioplankton communities in the Pearl River were Proteobacteria and Actinobacteria, which accounted for more than half the total abundance. This finding is consistent with previous river-based studies [17,40,41,42,43]. Proteobacteria play an important role in aquatic carbon and nitrogen cycling [44] and are dominant microorganisms in freshwater environments [42]. In addition, Actinobacteria can adapt well to various freshwater ecosystems due to pronounced ecophysiological plasticity [45]. Some taxa in Actinobacteria can utilize glucose and function in heterotrophic nitrification, thus playing vital roles in nutrient and energy cycling in aquatic habitats [46]. Ecological functions may explain the wide distribution of these groups in aquatic ecosystems.

Comparison of the bacterioplankton community structure among different tributaries of the Pearl River demonstrated significant spatial differentiations in flood season. Our results corroborated previous findings that bacterioplankton communities are highly dynamic along the longitudinal gradient of rivers [7,8,17]. Although no significant differences were found in species richness indices (Chao and ACE) among the three tributaries, the diversity indices (Shannon and Simpson) showed marked spatial variation, indicating heterogeneity of bacterioplankton abundance distribution among the different tributaries. For example, some lineages belonging to Proteobacteria, such as Methylophilales (order), Methylophilaceae (family), *Candidatus_Methylopumilus* (genus), and *Dechloromonas* (genus) were mainly restricted to PRD (LEfse; Figure 6). Genera such as *Deinococcus*, *Paenibacillus*, *Flavobacterium,* and *lubricus_group* were enriched in XJ, whereas *SM1A02* and *CL500_3* showed relatively high abundance in BJ. Further ordination analysis revealed that the BJ group was separated from the two other groups, whereas XJ and PRD showed partial overlap (Figure 5), despite the fact that the PRD is tidally influenced [19]. We therefore concluded that the bacterioplankton community structure in the PRD had obvious characteristics of freshwater ecosystems as this section lacked salinity. Similar results have also been reported in tidal reach areas of the Yangtze River [41]. 

Bacterioplankton communities are relatively sensitive to environmental perturbations, and environmental conditions and nutrient sources often determine microbial community composition among aquatic ecosystems [4,47]. Spatial variability can reflect changes in key environmental factors that influence the growth and survival of microbes [8]. In this study, the spatial patterns of bacterioplankton communities were similar to the cluster patterns of environmental factors. Mantel and RDA analyses showed that both physicochemical variables and land use significantly impacted the bacterioplankton communities, and together explained 57.6% of total variation. These results indicate that physicochemical variables and land use are dominant factors driving bacterioplankton communities in the Pearl River. Forward selection analysis demonstrated that SpC, DO, agricultural land, NH_4_-N, urban land, and water transparency were the most significant environmental variables influencing the Pearl River bacterioplankton communities. Both NH_4_-N and water transparency are reflections of the nutrient state in water bodies. Previous studies have identified the nutrient state as a leading factor affecting microbes [8,48,49], as the availability of nutrients may limit bacterioplankton growth in aquatic environments [50]. Nutrients can also influence phytoplankton and zooplankton compositions [51], and thus indirectly drive changes in river bacterioplankton communities via food web dynamics involving both phyto- and zooplankton. DO is considered an important index that affects the survival and reproduction of bacteria in aquatic ecosystems [52], and thus the structure of bacterioplankton communities is intimately linked to DO. We also observed that DO was the largest factor affecting the multifunctional diversity of the bacterioplankton communities. The significant role of SpC in shaping the bacterioplankton communities may partly be explained by its strong correlations with DO, water transparency, and NH_4_-N (Figure 2C) or other factors not measured in this study, as the effects of conductivity in freshwater rivers may not be directly due to mineral composition or the salts. Ruiz-González et al. also observed that conductivity is a primary environmental determinant for the bacterioplankton community in the Ebro River [53].

Anthropogenic factors such as agricultural and urban land use significantly contributed to the variability in the bacterioplankton communities among the three tributaries. This result may be explained by both direct and indirect anthropogenic influences on bacterioplankton community compositions. Firstly, bacteria may enter riverine waters directly through a variety of point and non-point sources such as urban runoff, wastewater treatment plant outflows, and agricultural areas [54,55]. The current study region is a major hub of China’s economic growth and one of the most urbanized areas in the world, with the Pearl River being the main water source for domestic, agricultural, and industrial usage in the surrounding cities [56]. Pollutants from agriculture, industrial, and manufacturing discharge, and municipal sewage cause serious environmental problems [57,58,59]. Therefore, it is not surprising that agricultural and urban land use significantly shaped the bacterioplankton communities in this study. Secondly, the shared effects of physicochemical factors and land use were 6.1%, suggesting that land use could structure the riverine bacterioplankton community via changing river conditions caused by water outflow [8]. In the current study, we observed a positive correlation between urban or agricultural land use and several physicochemical factors (e.g., NO_2_-N, TP, PO_4_-P, and SS). NO_2_-N, TP, and PO_4_-P are essential nutrients for primary production and microbial growth [60,61]. Suspended solids can act as a nutrient source for aquatic microorganisms and provide habitat for bacterial growth [4]. Therefore, land use activities could affect survival and growth of certain bacteria via these physicochemical factors, and thereby indirectly shape the bacterioplankton community. However, the pure effect of land use was only 8.9%, which was much smaller than that of physicochemical factors (42.6%). Our results indicate that, although land use matters, physicochemical variables are still the dominant factors structuring the bacterioplankton communities in this large river. In addition, since there are likely to be vertical gradients of physicochemical variables with depth, such as nutrients and particulate loads (suspended solids), it will be necessary to consider both vertical and horizontal patterns of bacterioplankton communities in future studies.

## 5. Conclusions

This study investigated the bacterioplankton communities in July 2018 in three main tributaries of the Pearl River, i.e., Beijiang, Xijiang, and Pearl River Delta, based on 16S rRNA high-throughput sequencing. We revealed that the three tributaries formed distinct bacterioplankton communities in relation to the environmental gradients. Both physicochemical factors and land use had direct effects on the bacterioplankton communities, and land use may shape bacterioplankton communities through indirect effects of physicochemical factors on the riverine ecosystem. However, physiochemical factors outweighed land use in structuring bacterioplankton communities in this large subtropical river. This study provided fundamental information on the diversity, spatial patterns, and influencing factors of bacterioplankton communities in the Pearl River, which should enhance our understanding of how bacterioplankton change in response to environmental gradients and anthropogenic activities. However, as the current study only covered one season, future studies are needed to provide insight into microbial communities and functions at both the spatial and temporal scale, including biological factors and other physicochemical variables.

## Figures and Tables

**Table 1:** Analysis of similarity (ANOSIM) test of bacterioplankton communities based on Bray–Curtis distances among three tributaries of the Pearl River.

Tests	Group	*R* Value	*p* Value
Global test	BJ–XJ–PRD	0.835	0.001
Paired comparison	BJ–XJ	0.998	0.001
	BJ–PRD	0.979	0.001
	XJ–PRD	0.385	0.001

## Supplementary Materials

The following are available online at https://www.mdpi.com/2076-2607/8/6/814/s1, Figure S1: Rarefaction curves of the different samples, Table S1: Differences of all predicted pathways in KEGG level 2 among tributaries using the Kruskal–Wallis test.

## Abbreviations

XJ	Xijiang River
BJ	Beijing River
PRD	Pearl River Delta
DO	Dissolved oxygen
TDS	Total dissolved solids
SpC	Specific conductivity
ORP	Oxidation-reduction potential
NH_4_-N	Ammonium
NO_3_-N	Nitrate
NO_2_-N	Nitrite
TP	Total phosphorus
PO_4_-P	Phosphate
SS	Suspended solids
OTU	Operational taxonomic units
PCA	Principal component analysis
NMDS	Nonmetric multidimensional scaling
ANOSIM	Analysis of similarity
LDA	Linear discriminant analysis
LEfSe	Linear effect size analysis
RDA	Redundancy analysis
VPA	Variation partitioning analysis
PICRUSt	Phylogenetic investigation of communities by reconstruction of unobserved states
