# Supplementary material for: Spatial Variation in Bacterioplankton Communities in the Pearl River, South China: Impacts of Land Use and Physicochemical Factors
Source: Microorganisms. 2020 May 29;8(6):814. doi: 10.3390/microorganisms8060814 (PMC7356326; doi:10.3390/microorganisms8060814)
Supplement: Supplementary file 1 [file microorganisms-08-00814-s001.pdf]

Spatial Variation in Bacterioplankton Communities in the Pearl River, South China: Impacts of Land Use and Physicochemical Factors

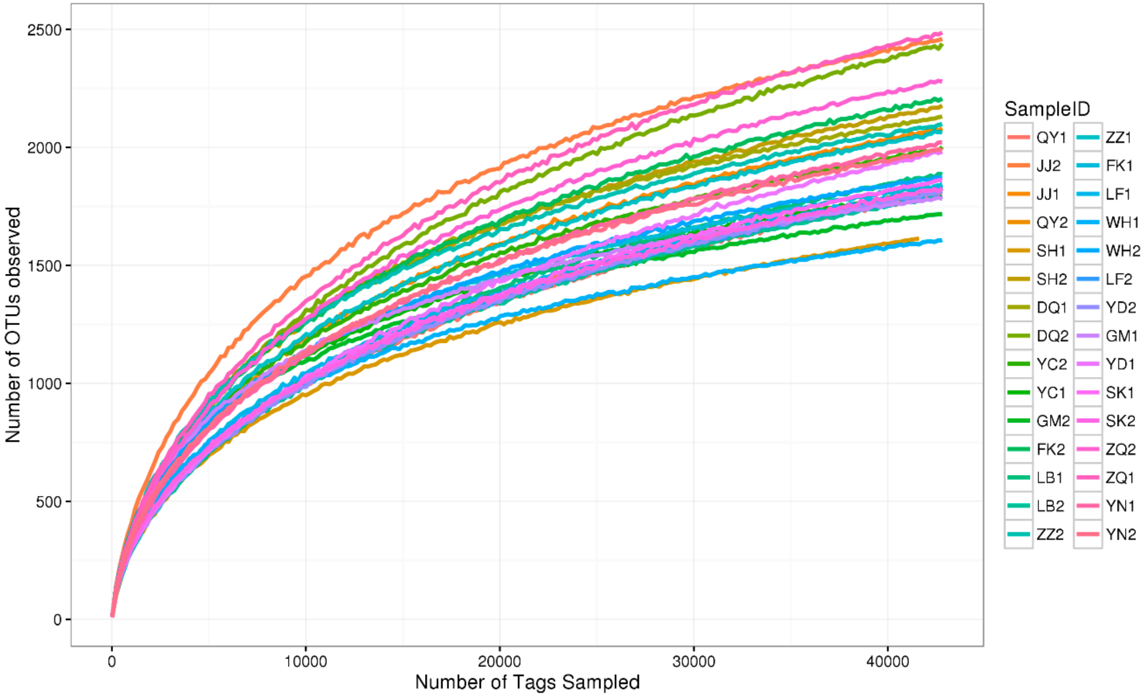

Figure S1. Rarefaction curves of the different samples.

Table S1. Differences of all predicted pathways in KEGG level 2 among tributaries using the Kruskal–Wallis test.

Level_1	Level_2	BJ	XJ	PRD	p-value	q-value
Metabolism	Amino Acid Metabolism	17108.5	15109.7	15869.4	1.17E-05	9.01E-05
Metabolism	Carbohydrate Metabolism	17544	14514.4	15283.6	8.81E-06	9.01E-05
Metabolism	Energy Metabolism	10833.3	10033.8	9698	0.00477	0.005565
Metabolism	Metabolism of Cofactors and Vitamins	7629.9	6861.3	6835.7	0.000146	0.000256
Metabolism	Lipid Metabolism	6429.7	5636.5	5928.8	8.81E-06	9.01E-05
Metabolism	Xenobiotics Biodegradation and Metabolism	5497.5	5145.1	5966.6	0.002115	0.002742
Metabolism	Nucleotide Metabolism	5332.7	4707.7	4735.6	5.98E-05	0.000201
Metabolism	Metabolism of Terpenoids and Polyketides	3669.5	3342	3501.4	0.000518	0.000824
Metabolism	Glycan Biosynthesis and Metabolism	3461.2	2902.3	2845.9	7.18E-05	0.000201
Metabolism	Enzyme Families	3286.9	2918.8	2928	9.18E-05	0.000201
Metabolism	Metabolism of Other Amino Acids	2997.8	2669.2	2852.6	4.34E-05	0.000201
Metabolism	Biosynthesis of Other Secondary Metabolites	1700.6	1501.9	1474.4	0.000185	0.000308
Genetic Information Processing	Replication and Repair	11311.3	10277.7	10521.3	6.86E-05	0.000201
Genetic Information Processing	Translation	7067.5	6412.8	6419.6	5.88E-05	0.000201
Genetic Information Processing	Folding, Sorting and Degradation	4054.9	3623.5	3626.1	6.26E-05	0.000201
Genetic Information Processing	Transcription	4308.3	3329	3450.8	1.29E-05	9.01E-05
Environmental Information Processing	Membrane Transport	19970.9	15320.4	16685.1	6.74E-06	9.01E-05
Environmental Information Processing	Signal Transduction	3629.9	3215.8	3515.6	0.002824	0.003408
Environmental Information Processing	Signaling Molecules and Interaction	271.4	255.4	268.5	0.000787	0.001102
Cellular Processes	Cell Motility	3693.2	3910.8	4425.6	0.019867	0.021729
Cellular Processes	Cell Growth and Death	727.3	726.4	728.3	**0.999032**	0.999032
Cellular Processes	Transport and Catabolism	566.6	521.5	520.8	0.000701	0.001022
Human Diseases	Infectious Diseases	685	582.4	568.9	0.000118	0.000229
Human Diseases	Neurodegenerative Diseases	350.4	361.9	404.1	0.026748	0.028369
Human Diseases	Cancers	222.1	199	195.4	0.000108	0.000223
Human Diseases	Metabolic Diseases	175.7	159.8	148.2	0.000646	0.000983
Human Diseases	Immune System Diseases	79.7	72.3	81.6	0.007556	0.008531
Human Diseases	Cardiovascular Diseases	5.9	8	8.3	8.51E-05	0.000201
Organismal Systems	Endocrine System	653.6	660.9	710.9	0.002363	0.002954
Organismal Systems	Environmental Adaptation	232.6	208.3	217.1	0.000141	0.000256
Organismal Systems	Nervous System	153	136.4	131.6	0.001404	0.00189
Organismal Systems	Immune System	92.8	81.4	80.4	8.74E-05	0.000201
Organismal Systems	Digestive System	67.2	71.3	65.4	**0.409709**	0.421759

Organismal Systems	Excretory System	72.7	57.6	60.8	7.59E-05	0.000201
Organismal Systems	Circulatory System	34.2	51.4	66.8	5.91E-05	0.000201
